# NOVEL AGING BIOMARKERS IN THE HRS

**DOI:** 10.1093/geroni/igz038.1611

**Published:** 2019-11-08

**Authors:** Eileen Crimmins

**Affiliations:** Davis School of Gerontology, University of Southern California, Los Angeles, California, United States

## Abstract

In addition to the broad panel of aging related biomarkers available in HRS, we will describe measurement of novel aging biomarkers such as telomere length and mitochondrial DNA copy number in 4000 HRS participants. Both these biomarkers were measured in DNA obtained from whole unsorted blood using quantitative real time polymerase chain reaction (PCR) and were adjusted for individual cell composition measured from flow cytometry. We will describe the relationship between these two biomarkers and other measures of biological age available in HRS. Differences in these two novel aging biomarker by socioeconomic status, race/ethnicity, and exposure to early life hardships will be presented to clarify the value of the data to further unravel how social factors get under the skin to affect the process of aging.

